# Comparative Morphology, Transcription, and Proteomics Study Revealing the Key Molecular Mechanism of Camphor on the Potato Tuber Sprouting Effect

**DOI:** 10.3390/ijms18112280

**Published:** 2017-10-30

**Authors:** Li-Qin Li, Xue Zou, Meng-Sheng Deng, Jie Peng, Xue-Li Huang, Xue Lu, Chen-Cheng Fang, Xi-Yao Wang

**Affiliations:** 1 College of Agronomy, Sichuan Agricultural University, Chengdu 611130, China; liliqin@sicau.edu.cn (L.-Q.L.); zou_xue_2008@aliyun.com (X.Z.); dengmengsheng87@hotmail.com (M.-S.D.); gusongke1988@163.com (J.P.); hxueli1983@163.com (X.-L.H.); lorilu0917@163.com (X.L.); chenchengfang0211@163.com (C.-C.F.); 2 Mianyang Academy of Agricultural Sciences, Mianyang 621023, China

**Keywords:** potato, tuber, sprouting, camphor, transcription, proteomics

## Abstract

Sprouting regulation in potato tubers is important for improving commercial value and producing new plants. Camphor shows flexible inhibition of tuber sprouting and prolongs the storage period of potato, but its underlying mechanism remains unknown. The results of the present study suggest that camphor inhibition caused bud growth deformities and necrosis, but after moving to more ventilated conditions, new sprouts grew from the bud eye of the tuber. Subsequently, the sucrose and fructose contents as well as polyphenol oxidase (PPO) activity were assessed after camphor inhibition. Transcription and proteomics data from dormancy (D), sprouting (S), camphor inhibition (C), and recovery sprouting (R) samples showed changes in the expression levels of approximately 4000 transcripts, and 700 proteins showed different abundances. KEGG (Kyoto encyclopaedia of genes and genomes) pathway analysis of the transcription levels indicated that phytohormone synthesis and signal transduction play important roles in tuber sprouting. Camphor inhibited these processes, particularly for gibberellic acid, brassinosteroids, and ethylene, leading to dysregulation of physiological processes such as cutin, suberine and wax biosynthesis, fatty acid elongation, phenylpropanoid biosynthesis, and starch and sucrose metabolism, resulting in bud necrosis and prolonged storage periods. The KEGG pathway correlation between transcripts and proteins revealed that terpenoid backbone biosynthesis and plant–pathogen interaction pathways showed significant differences in D vs. S samples, but 13 pathways were remarkably different in the D vs. C groups, as camphor inhibition significantly increased both the transcription levels and protein abundance of pathogenesis-related protein PR-10a (or STH-2), the pathogenesis-related P2-like precursor protein, and the kirola-like protein as compared to sprouting. In recovery sprouting, these genes and proteins were decreased at both the transcriptional level and in protein abundance. It was important to find that the inhibitory effect of camphor on potato tuber sprout was reversible, revealing the action mechanism was similar to resistance to pathogen infection. The present study provides a theoretical basis for the application of camphor in prolonging seed potato storage.

## 1. Introduction

Potato (*Solanum tuberosum* L.) is the fourth most important food crop worldwide, which is a reflection of its high yield, extensive adaptability, starch content, substantial amounts of essential vitamins, and low fat content. Compared to grain food crops, such as rice, wheat, and corn, the storage time of potato tubers, which have a high moisture content (approximately 80%), is relatively short because tubers will sprout after cessation in dormancy (between 30 and 150 d at room temperature for different varieties). Tubers can be roughly divided into seed and commodity potatoes. If the planting season is much later than the time of seed germination, the yield will be reduced as a result of premature aging of the tubers [1]. Fresh or industrially processed tubers show a reduction or loss of their commercial value because of sprouting. Therefore, control of tuber dormancy using physical, chemical, or genetic methods is critical for potato storage. Dormancy and sprouting comprise a complex set of physiological processes that are regulated by endogenous hormones, such as ABA (Abscisic acid), a major hormonal regulator of the initiation and maintenance of dormancy. By contrast, gibberellins and cytokinins are likely involved in bud dormancy release [2].

Tuber dormancy is closely associated with genotype, and in a certain range, low temperature (2–5 °С) conditions can extend tuber dormancy but not stop sprouting; thus, the use of sprout inhibitors is necessary to extend the storage period for potato tubers. Chlorpropham (isopropyl *N*-3-chlorophenyl carbamate; CIPC), a sprout inhibitor that is applied post-harvest, is used globally to prevent the sprouting of stored commodity potatoes. However, there is a decreasing market tolerance for agrochemical residues on food products [3].

Research has confirmed that 1,4-dimethylnaphthalene, a natural volatile product found in dormant tubers, induces expression of the cell cycle inhibitors KRP1 and KRP2 (Kip-related protein) to prevent bud growth, but tuber surface residues below 1.4–2.7 mg/kg do not suppress sprouting [4,5]. Essential oils extracted from some aromatic plants also display different degrees of inhibition of potato tuber sprouting. Essential oils from peppermint (*Mentha piperita* L.), coriander (*Coriandrum sativum* L.), and eucalyptus (*Eucalyptus globulus* Labill.) have shown variable degrees of tuber sprouting inhibition after treatment for 10 d, but limonene contained in the latter two oils could result in a large number of rotten tubers, whereas peppermint oil does not cause this phenomenon [6]. Although carvone, obtained from mint (*Mentha spicata* L.) essential oil, also inhibits sprouting in potato tubers as well as fungal and bacterial reproduction [7,8,9], this compound has dual effects: low concentrations promote sprouting, whereas high concentrations result in bud death [9].

High-throughput mRNA sequencing (RNA-seq) is a powerful tool for comparing gene expression [10]. Using RNA-seq analysis, Cheng et al. revealed that ethylene-mediated reproductive organ development and abscission in soybean were correlated to specific metabolite groups, such as plant hormone biosynthesis and signal transduction, starch and sucrose metabolism, and secondary metabolism [11]. Liu et al. identified 26,639 genes, including 5912 and 3885 differentially expressed genes from dormancy tuber (DT) vs. dormancy release tuber (DRT) and DRT vs. sprouting tuber (ST), respectively, using RNA-seq. Moreover, these authors showed that dormancy release was accompanied by stress response and redox regulation [12]. Isobaric tag for relative and absolute quantitation (iTRAQ) is considered one of the most robust methods of differential quantitative proteomics analysis [13]. Yang et al. analysed the dynamics of protein expression associated with cold-induced sweetening in potatoes using an iTRAQ labelling strategy. In this study, a total of 4463 potato proteins were identified, of which 46 proteins showed differential expression during potato tuber cold storage [14]. Liu et al. identified 1752 proteins associated with tuber dormancy release after analysing dormant tuber (DT), dormancy release tuber (DRT) and sprouting tuber (ST) using iTRAQ technology [15].

Camphor (1,7,7-trimethyl-bicyclo [2.2.1] heptanes-2-one) is a volatile compound derived from the camphor tree. However, inhibitory effects of this compound on bud have not been assessed. In the present study, camphor regulated bud growth. Subsequently, bud resin sections were analysed, and sucrose, fructose content and polyphenol oxidase (PPO) activity were measured. To reveal the mechanism of camphor inhibition and enhance the current understanding of the tuber sprouting mechanism, we analysed transcription and proteomics data from four samples: dormancy, sprouting, camphor inhibition and recovery sprouting. Correlation analyses between transcripts and proteins were performed to characterize the gene and protein regulatory networks underlying the effect of camphor on potato tuber sprouting.

## 2. Results

### 2.1. Flexible Inhibition of Tuber Sprouting Using Camphor

**Figure 1:**
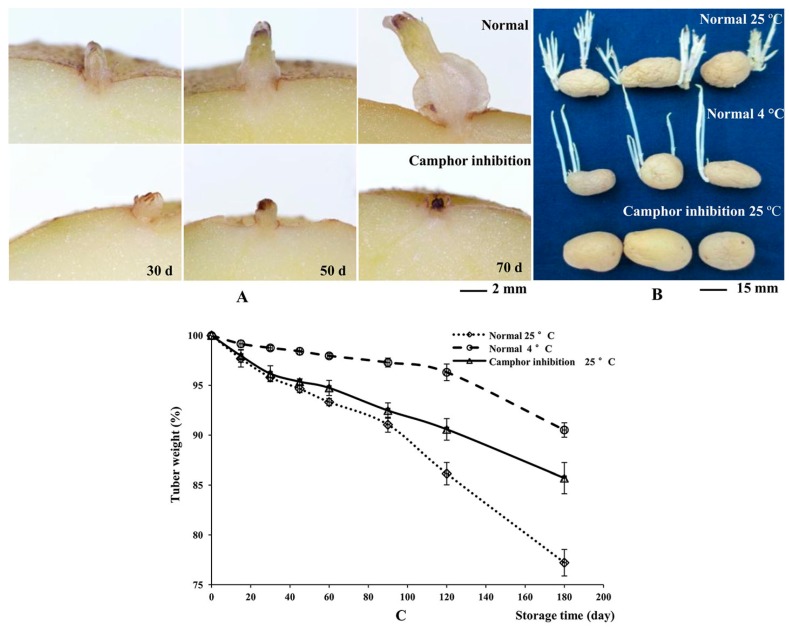
The effects of camphor inhibition on sprouting and tuber weight during storage. (**A**) Contrast of tuber sprouting between normal and camphor inhibition; (**B**) Morphological comparison of tubers stored under different conditions at 180 d; (**C**) Tuber weight loss during storage.

Minitubers were divided into two groups and placed into boxes in the dark at 23 ± 2 °С. The tuber buds grew 2–10 mm from 30 to 70 d, whereas after camphor inhibition for 50 d, the top of the bud was blackened, revealing necrosis. All of the buds died after treatment for 70 d (Figure 1A). Under 25 °С storage conditions for 180 d, the tuber surface showed significant shrinkage and weight loss of 22.78%, because of the growth of buds and related nutrient consumption (Figure 1B,C). Under 4 °С low temperature conditions, tuber metabolic activity was weak, and only a 9.48% weight loss was observed. No bud growth was observed for camphor-treated tubers, with no surface shrinkage phenomenon and only half of the weight loss observed under the 25 °С storage conditions.

**Figure 2:**
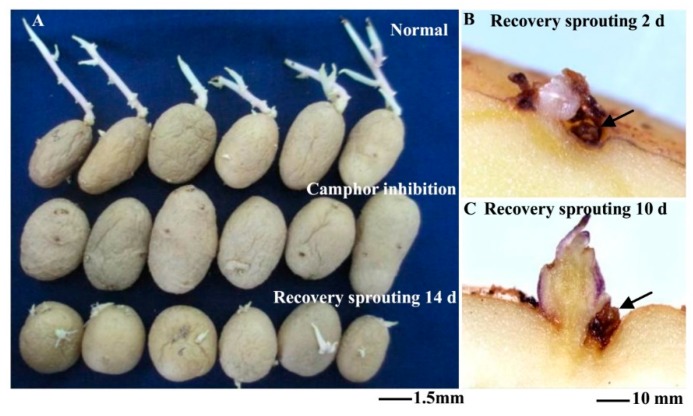
Recovery sprouting after moving to ventilation conditions for 2 d, 10 d and 14 d. (**A**) Comparison of shoot growth under different treatments; (**B**) New sprout growth from the bud eye region after recovery sprouting for 2 d; (**C**) New sprout growth from the bud eye region after recovery sprouting for 10 d. Arrows indicated the original dead bud tissue.

After storage for 110 d, the tubers were treated and moved to ventilation conditions to remove camphor. Subsequently, all tubers sprouted at 14 d after recovery (Figure 2A). The shoot length of control tubers was approximately 30.65 mm, which was too long for sowing, and the shoot length of tubers after recovery sprouting was approximately 8.64 mm, which was conducive for sowing. At the same time, new sprouts grew from the bud eyes were observed at 2 d and 10 d after recovery (Figure 2B,C). The results of pot planting showed that the number of seeding and tubers per pot were significantly increased after recovery sprouting compared to the control group, but the tubers were small, reflecting decreased yield (Table 1). Camphor-treated tubers could restore sprouting after storage for 180 d, but the new buds did not develop to the leaves and roots and showed a loss of morphogenetic potential. These results showed that although tubers could recover sprouting after camphor inhibition, if the treatment time was too long (over 110 d), plant growth and tuberization were affected.

### 2.2. Effects of Camphor Inhibition on Histological Structures in the Bud Eye Region 

**Figure 3:**
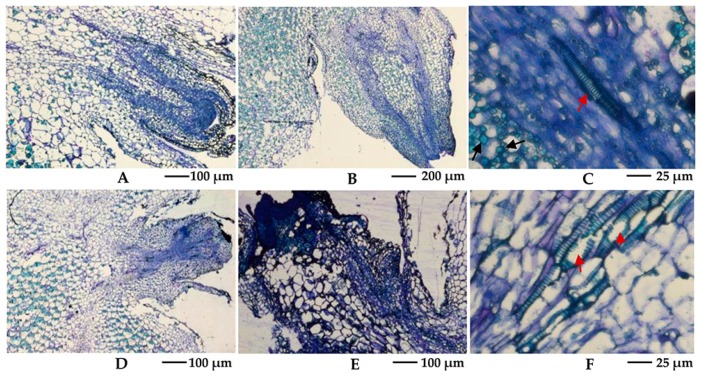
Comparison of the histological structures in the tuber bud between camphor inhibition and normal conditions. (**A**–**C**) Histological structures in the normal bud: (**A**) A bud of approximately 0.5 mm; (**B**) A shoot of approximately 2 mm; (**C**) A vessel in the vascular tissue (red arrows) and starch grains in cell (black arrows). (**D**–**F**) Histological structures in the bud after camphor inhibition: (**D**) Malformed buds lacking an apical meristem; (**E**) Necrotic sprouts and cell vacuolization; (**F**) Vessel breakage in the vascular tissue (red arrows), and loss of starch grains.

Harvested tubers were cured for 14 d and stored under 25 °С conditions. Buds of approximately 2 mm were obtained after storage under normal conditions for approximately 50 d, whereas the shoot tip showed blackening after camphor inhibition. Histological structures were observed using a stereomicroscope, and the results suggest that camphor inhibition leads to bud growth deformities, with no formation of the apical meristem region. During treatment, the top of the bud could not grow and became necrotic, with vacuolization of the internal cells (Figure 3B,E). In the mid-term, the vessels in the tuber, particularly the areas connecting the bud and tuber flesh, were damaged, and intracellular starch grains disappeared after camphor inhibition (Figure 3C,F). This phenomenon may hinder access to the nutrient supply, further reducing the resistance of the bud to external stress.

### 2.3. Analysis of Sucrose and Fructose Content and PPO Activity

**Figure 4:**
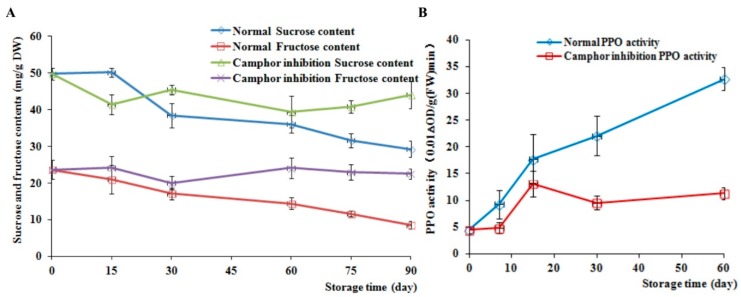
Effects of camphor inhibition on the sucrose and fructose contents as well as polyphenol oxidase (PPO) activity in tubers. (**A**) The contents of sucrose and fructose; (**B**) The PPO activity.

The sucrose and fructose contents were measured every 15 d after storage. A total of six points were assessed, and the PPO activity was measured every 10 d after storage. The sucrose and fructose contents in the control decreased by 41.19% and 64.13%, respectively, at 90 d compared to 0 d (Figure 4A), whereas after camphor inhibition, the sucrose and fructose contents fluctuated during storage. During sprouting of normal tubers, metabolic activity was vigorous and the activity of PPO displayed a sharp increase (Figure 4B). However, the PPO activity in treated tubers was elevated between 10 and 15 d, followed by a decrease to steady state. The difference between the control and camphor inhibition reached a maximum after 60 d, where the former was 2.89-fold higher than the latter at this time point.

### 2.4. Evaluation of Differentially Transcribed Genes

Camphor has the potential to extend seed potato storage, reflecting its effective sprouting inhibition, and restore sprout growth through treatment at a low concentration. To explore the mechanism underlying the effect of camphor on tuber sprouting at the gene expression level, 10.97, 11.83, 12.06, and 12.29 million sequenced tags for the dormancy, sprout, camphor inhibition and recovery sprout, respectively, were obtained, using Illumina HiSeq^TM^ 2000 sequencing. Compared to the potato reference genome, the unique match reads were 7,841,403, 8,463,619, 8,209,691, and 8,660,621, respectively.

With the use of the dormant library (D) as a control, comparative analysis of the sprouting (S), camphor inhibition (C), and recovery sprouting (R) libraries revealed 3705, 3151, and 2578 up-regulated genes and 222, 883, and 468 down-regulated genes, respectively; 208 and 90 genes were up-regulated and 542 and 537 genes were down-regulated in the camphor inhibition and recovery sprouting samples, respectively, compared with the S conditions. We also identified 146 up-regulated genes and 360 down-regulated genes in recovery sprouting compared with camphor inhibition, and all differentially expressed gene (DEG) information was listed (Tables S1–S5). Based on the numbers of differentially expressed genes and the ratio of up/down-regulated genes, we concluded that the gene expression profiles of C, R and S were similar to each other but different from the profile of D. A total of 12 genes were randomly selected to validate the transcription sequencing results using quantitative real-time PCR. The expression patterns of the 12 genes in the four samples showed similarity between the two experiments, indicating good consistency (Figure S1).

### 2.5. KEGG Pathway Enrichment Analysis of DEGs

**Figure 5:**
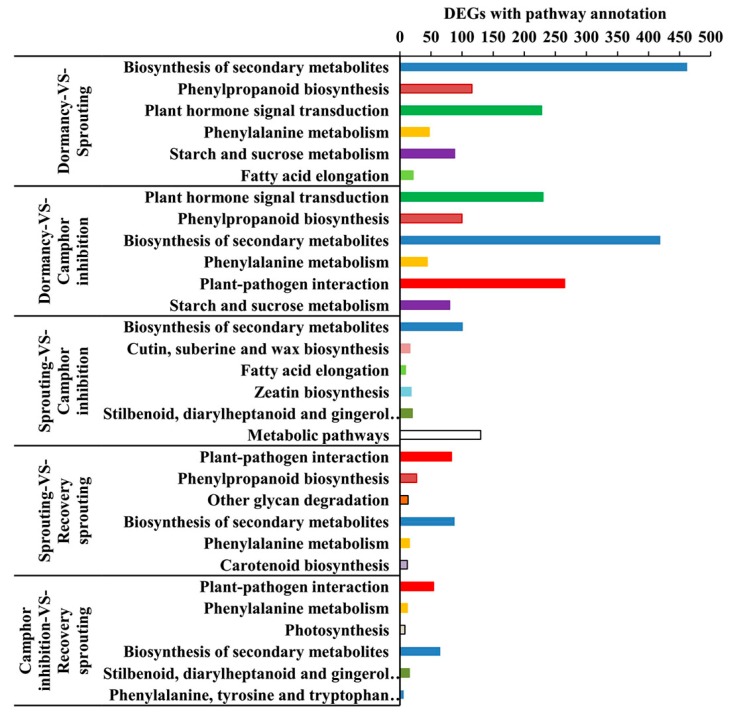
The first six pathways for differentially expressed genes (DEGs) in different comparison groups.

To obtain additional information for the DEGs, the biological metabolic pathways were investigated using enrichment analysis. In D vs. R, it was shown that 2396 DEGs were enriched in 122 metabolic pathways and approximately 24 pathways were significant at a *Q* value <0.05. There were approximately 16, 30, 12, 16, and 14 significantly enriched pathways in D vs. C group, D vs. R group, S vs. C group, S vs. R group, C vs. R group, respectively. The first six pathways in five different comparisons were illustrated (Figure 5). Compared to dormant samples, the enriched pathways of sprouting vs. camphor inhibition were similar, including plant hormone signal transduction, biosynthesis of secondary metabolites, phenylpropanoid biosynthesis and phenylalanine metabolism, and starch and sucrose metabolism. Camphor inhibition up-regulated gene expression associated with plant–pathogen interaction pathways and down-regulated gene expression associated with the cutin, suberine, and wax biosynthesis pathways and fatty acid elongation.

### 2.6. Typical Expression Patterns of DEGs under Four Samples

According to the expression pattern of DEGs in dormancy, sprouting, camphor inhibition, and recovery sprouting samples, the DEGs can roughly be divided into five categories. Representative genes from each of the five typical expression patterns are shown (Table 2). Most of DEGs belonging to type A and type B genes (approximately 3000), expressed at high levels in sprouting tubers, were significantly inhibited by camphor to various degrees, and subsequently, after recovery sprouting for 3 days, the expression of these genes increased. Type A and B genes were associated with growth and development, including transcription factors, kinases, and other regulatory factors. Approximately 200 DEGs belonging to type C, which had the highest expression levels in dormancy, may be associated with maintaining tuber dormancy. The expression of type D genes was similar in dormancy and sprouting, but the expression levels dramatically decreased after camphor inhibition and subsequently increased when camphor was removed. Speculatively, these genes may be related to the maintenance of basic life activities. Meristem death observed after camphor inhibition may reflect the low expression level of these genes, and plant stress resistance genes (Type E), such as the wound-induced protein and osmotin, were induced after camphor inhibition, implicating the involvement of these genes in the high resistance of tubers facing camphor damage.

### 2.7. Analysis of Differentially Abundant Proteins (DAPs)

Quantitative proteomics-based iTRAQ revealed 117 proteins with increased abundance and 159 proteins with decreased abundance in the D vs. S groups. A total of 285 proteins with increased abundance and 183 proteins with decreased abundance were detected in the S vs. C group, while 136 proteins with increased abundance and 140 proteins with decreased abundance were observed in the C vs. R group. Moreover, 361 proteins with increased abundance and 292 proteins with decreased abundance were detected in the D vs. C groups, and 325 proteins with increased abundance and 244 proteins with decreased abundance were detected in the S vs. R groups. In order to verify the accuracy of quantitative proteomics data, a total of 11 proteins were randomly selected and expression of corresponding genes were confirmed by quantitative RT-PCR. The results suggested eight gene expression patterns in different comparisons showed the same tendencies with respect to protein abundance. The primer sequence and the result of quantitative RT-PCR are listed in Table S6 and Figure S2.

**Figure 6:**
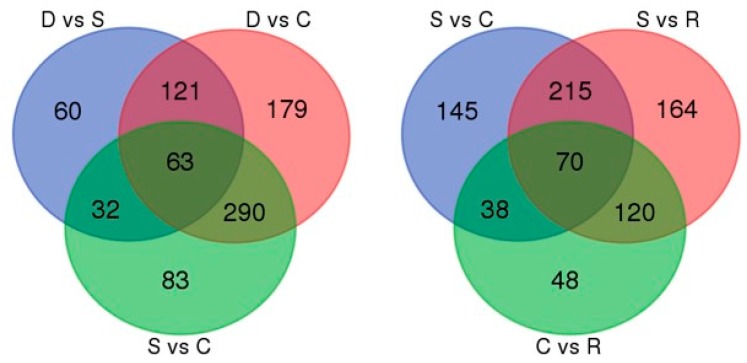
Venn diagram showing the numbers of differentially abundant proteins (DAPs) in various comparative groups.

**Figure 7:**
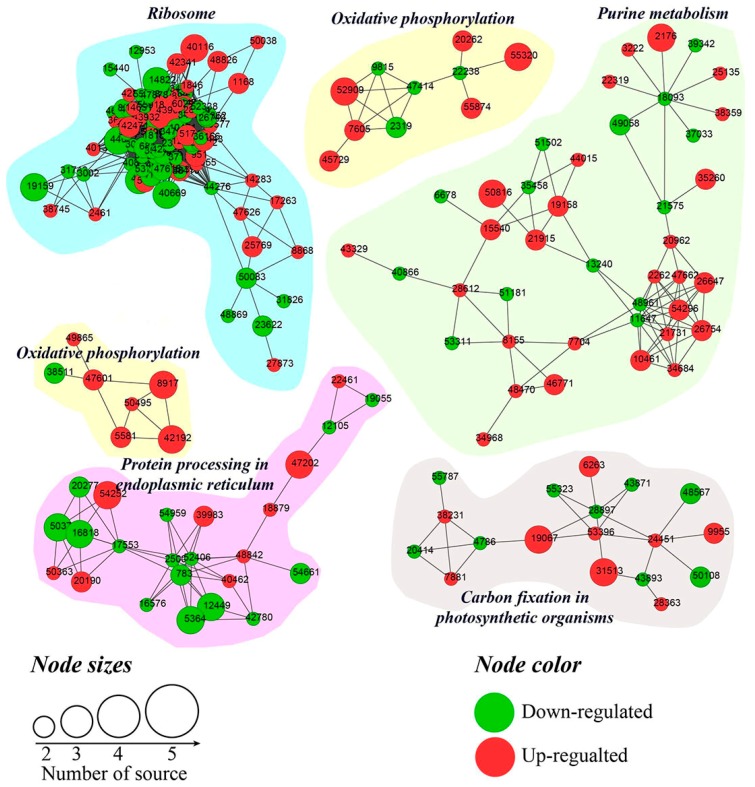
Functional correlation network of DAPs.

A Venn diagram of the numbers of DAPs in comparative groups illustrated 63 and 70 co-expressed DPAs among D-S-C groups and S-C-R groups, respectively (Figure 6). For more information see Figure S3. A total of 16 co-expressed DAPs among five comparative groups were found, including glycine-rich RNA-binding protein GRP1A, 26S proteasome non-ATPase regulatory subunit 10, and WRKY transcription factor 20 showed increased abundance, while eukaryotic translation initiation factor 5A-5, a 17.4 kDa class III heat shock protein, and polygalacturonase inhibitor-like showed decreased abundance. The protein–protein interaction network identified 193 DAPs that primarily belong to ribosome, purine metabolism, oxidative phosphorylation, protein processing in the endoplasmic reticulum, and carbon fixation in photosynthetic organism pathways. Many proteins from the interaction network belonged to ribosome and purine metabolism pathways (Figure 7). Information for all of the proteins identified in the interaction networks is shown (Table S7).

### 2.8. Correlation Analysis between Transcription and Proteomics

A correlation analysis to examine the consistency between transcription and proteomics revealed 15 genes and proteins with the same trends in the D vs. S group, 12 genes and proteins in the S vs. R group, and 5 genes and proteins in the C vs. R group. In the D vs. S group, approximately 290 genes showed notable changes in expression, while the corresponding proteins showed no changes. Additionally, 219 proteins showed notable changes in abundance, while the corresponding genes showed no changes. In the S vs. C group, 38 genes showed notable changes in expression levels, with the corresponding proteins showing no changes. By contrast, 349 of the proteins showed notable changes in abundance, while the corresponding genes showed no changes. A total of 25 genes showed notable changes in expression, with the corresponding proteins showing no changes in the C vs. R group. All results of correlation analysis were listed (Table S8).

**Figure 8:**
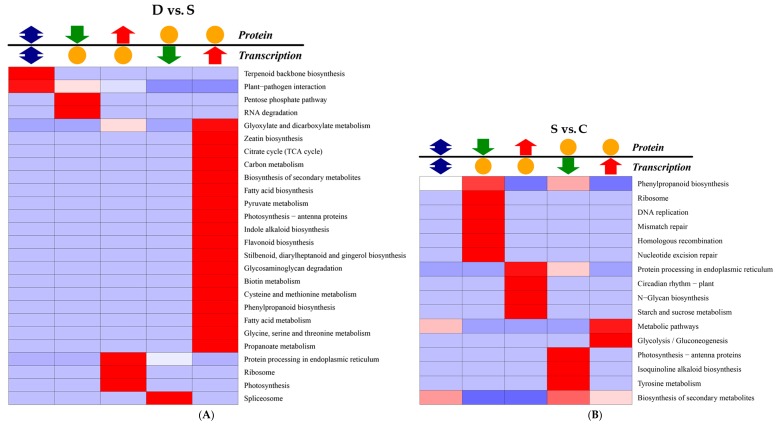

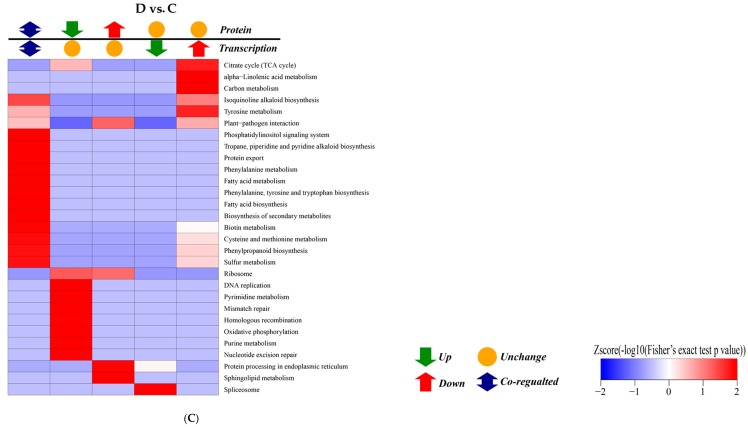
Enrichment correlation analyses of KEGG pathway in various comparative groups. Note: (**A**) KEGG pathway between transcription and proteomics in the D vs. S group; (**B**) KEGG pathway in the S vs. C group (**C**) KEGG pathway in the D vs. C group. There were simultaneous changes in both protein and transcription levels. KEGG, Kyoto encyclopaedia of genes and genomes.

Correlation analysis of the KEGG pathway between transcription and proteomics suggested that terpenoid backbone biosynthesis and the plant–pathogen interaction pathway showed significant differences in the D vs. S group (Figure 8A); No notable differences in these pathways were observed in the S vs. C group (Figure 8B), however, 13 pathways were obviously different in the D vs. C group (Figure 8C), with phenylpropanoid biosynthesis, biotin metabolism, and fatty acid biosynthesis showing the most significant differences. Correlation analysis of the KEGG (Kyoto encyclopaedia of genes and genomes) pathway in C vs. R group and S vs. R group were shown (Figures S4 and S5).

## 3. Discussion

### 3.1. Insight into the Transcription and Proteomics Analysis for Dormancy vs. Sprouting

**Figure 9:**
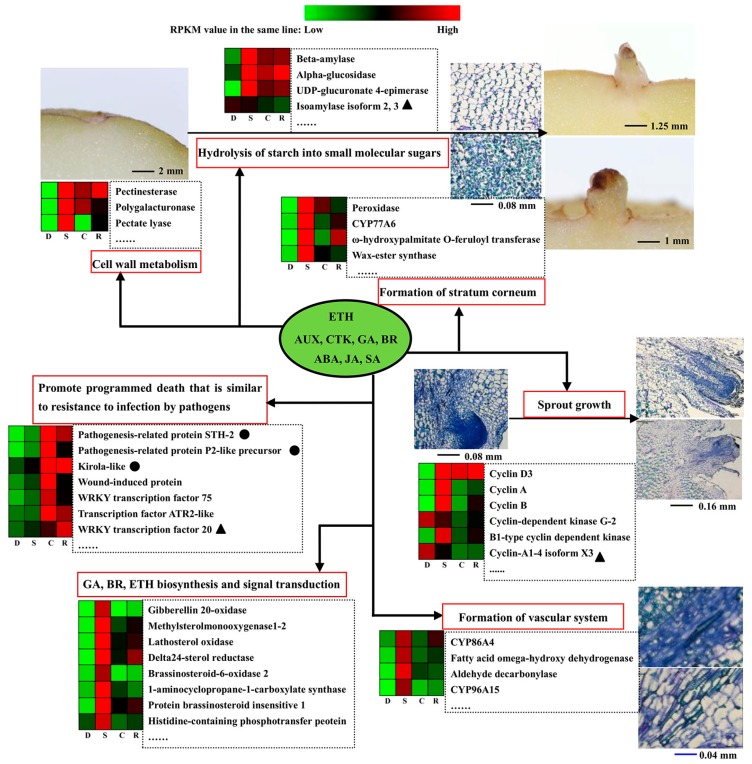
The major genes and proteins involved in tuber dormancy, sprouting and the camphor inhibition. Protein with circle exhibited the same tread at transcriptional and translational levels and the heatmap production using transcription RPKM (Reads per Kb per million reads) values; Protein with triangle meant that changes occurred in translational level; Protein without any symbol meant that changes occurred in transcriptional level.

Similar to the dormancy of conventional seeds, potato tuber dormancy is also co-regulated by genotype and environmental factors [16]. Potato tuber dormancy and sprouting refer to a series of perplexing physiological process, such as carbohydrate metabolism, protein and DNA synthesis, and signal transduction [17]. In the present study, correlation analysis of the KEGG pathway showed 15 genes and proteins with common trends in the D vs. S groups (Table S5), showing the enrichment of terpenoid backbone biosynthesis (farnesyl pyrophosphate synthase1-like, isopentenyl-diphosphate delta-isomerase I, and hydroxymethylglutaryl-CoA synthase-like) and plant–pathogen interaction pathways (calcium-binding protein CML13 and calmodulin) (Figure 9). Terpenoids are indispensable for the growth and development of plants, and the terpenoid biosynthesis pathway contributes to the synthesis of GA(Gibberellin) and BR(Brassinolide) [18,19] and plays important roles in the formation of carotenoids in photosynthesis and maintenance of the cell membrane structure and stability. Proteins with high abundance in the terpenoid pathway are likely required for dormancy release progression in potato tubers [20].

In previous studies, CMLs (Calmodulin-like proteins) played important roles in plant growth, development and response to stress signals [21,22]. In the present study, calmodulin and the putative calcium-binding protein CML13 showed increased expression in D vs. S group by KEGG pathway enrichment. However, Liu et al. previously showed that calmodulin was down-regulated in sprouting tubers [23]. This discrepancy may reflect differences in plant variety and experimental conditions. Moreover, an ATP-dependent (S)-NAD(P)H-hydrate dehydratase showed a decrease in both the transcription level and protein abundance, and this enzyme, which has been implicated in osmotic and alcohol stress, plays a significant role in the survival of stressed cells through repairing metabolite enzymes [24]. Thus, the high expression abundance of proteins in dormancy may help tubers to survive under stress. SN2 mediates cell division, elongation, and antibacterial processes and has also been implicated in crosstalk between gibberellins, abscisic acid and brassinosteroids [25,26]. The transcription data revealed that this gene showed obviously increased expression levels in D vs. S group (Table 2), consistent with previous reports [12,23]. Thus, we speculate that SN2 plays an important role in maintaining the dormancy state of potato tuber.

### 3.2. Camphor Effect on Potato Tuber Sprouting

The results of the present study further demonstrated camphor inhibition of tuber sprouting to extend the storage period and recovery of sprouting in the bud eye region after removing camphor. The physiological state as well as transcription and quantitative proteomics profiles of camphor inhibition were similar to sprouting and significantly different from dormancy. We speculate that camphor inhibition does not prolong the normal dormant state, but rather inhibits sprout growth. These results were similar to those obtained with the sprouting inhibitors 1,4-dimethylnapthalene and chlorpropham, and the phosphatases and proteins associated with oxygen-related metabolism were up-regulated in tubers after treatment with these two sprout inhibitors [27]. In the present study, camphor inhibition also affected the expression level and abundance of genes and protein related to oxygen stress, but this effect was not prominent, as the most striking function of camphor was inhibition of cell epidermis formation and synthesis of cuticle material. Moreover, camphor, 1, 4-dimethylnapthalene, and chlorpropham affected the *CYCD* genes related to the cell division cycle [4], indicating that camphor undergoes a mechanism similar to that of 1,4-dimethylnapthalene and chlorpropham but also has its own characteristics.

Pathogenesis-related proteins (PRPs) are key indicators of acquired resistance [28]. The transcripts of potato STH-2 (Pathogenesis-related protein-10a, PR-10a) rapidly accumulate in response to treatment with the elicitor arachidonic acid, and a low concentration of spores induced this accelerating accumulation; therefore, STH-2 was classified as a PRP family protein [29]. However, transgenic potatoes over-expressing STH-2 showed no enhanced resistance to *Phytophthora infestans* or potato virus X [30,31,32]. In the present study, camphor inhibition caused a significant increase in both the STH-2 transcript (approximately 10 times) and the STH-2 protein (approximately 11 times) levels as compared to sprouting. In recovery sprouting, both the transcript and protein levels were decreased. Except for STH-2, other PRPs, such as pathogenesis-related protein P2-like precursor and kirola-like protein, showed similar changes in both transcript levels and protein abundance. We speculated that camphor inhibition might lead to a stress response similar to resistance to pathogen infection.

Carboxylesterase 120 had had same change trend with STH-2 in the S vs. C group, but in S vs. R groups, the carboxylesterase 120 transcript and protein levels were also increased. Recent studies have suggested that this gene has a higher expression level in summer buds than during paradormancy in grapes [33]. Thus, carboxylesterase 120 may have a similar function in potatoes. Ahn and Zimmerman reported that transgenic potato lines overexpressing carrot HSP17.7 also exhibited enhanced tuberization in vitro under constant heat stress at 29 °C, suggesting this protein functions in tuber formation. In the present study, 17.7 kDa class I heat shock protein-like was decreased in the S vs. C groups and exhibited the same trend in the S vs. R groups; suggesting that this protein is involved in bud growth. These two proteins showed the same trends in the two comparative groups, suggesting that the effect of camphor on their expression patterns was irreversible, maybe because the recovery sprouting for 3 days was short.

A total of 24,407 transcripts was detected via transcription sequencing, whereas iTRAQ only detected 2500 proteins. Many proteins were not detected because of technical limitations, and it was not clear how these proteins changed. Thus, the transcriptome provides more information than the proteome. In the present study, KEGG pathway analysis of the transcription levels revealed that phytohormone synthesis and signal transduction played important roles in tuber sprouting. Liu et al. also showed that overexpressed genes, including auxin, gibberellic acid, cytokinins and brassinosteroids, were dominant and that various cyclin isoform-associated genes involved in cell division/cycle were primarily up-regulated in dormancy tuber vs. dormancy release tuber [12]. However, these pathways were intercepted or inhibited by camphor, and restored expression levels were observed in recovery sprouting (genes in type A or B) in our study.

Inhibition of camphor greatly damages the expression of genes encoding enzymes involved in cell wall specialization, which was the basis for forming vascular bundles and epidermis, such as fatty acid ω-hydroxy dehydrogenase, aldehyde decarbonylase, and wax-ester synthase. This result could explain the observed bud tissue and vascular system damage resulting from camphor inhibition in histological structures (Figure 4). This phenomenon was similar to the vascular tissue damage resulting from mint essential oil treatment [9]. Ibañes et al. showed that brassinosteroid signalling modulated the bundle number to establish the periodic pattern of Arabidopsis shoot vascular bundles by promoting early procambial division [34]. In the present study, expression levels of the methylsterol monooxygenase 1 (*SMO1*), delta (7)-sterol-5-desaturase (*ERG3*), delta 24-sterol reductase 1 (*DWF1*), brassinosteroid-6-oxidase 1 (*BR6OX1*), protein BR insensitive 1 (*BRI1*), BRsignalling kinase (*BSK*), brassinosteroid-resistant 1 (*BZR1*), cyclin D3 (*CYCD3*), and cyloglucosyl transferase (*TCH4*) genes, involved in brassinosteroid synthesis, signal transduction, and regulation, were inhibited during camphor treatment compared with sprouting. The block in brassinosteroid signalling may subsequently result in the obstruction of vascular bundle formation or repair. The transcription and activity of pectatelyase were increased in mango in response to ethylene treatment [35,36]. In the present study, expression of 1-aminocyclopropane-1-carboxylate synthase, the rate-limiting enzyme of ethylene biosynthetic pathway, was up-regulated, and pectate lyase showed increased expression during sprouting and decreased expression during camphor inhibition. The increased expression of pectatelyase contributed to cell wall degradation and integration of material into storage cells, which could promote the transport of substances. These findings suggest that during sprouting, ethylene synthesis is increased to promote the expression of genes related to cell wall degradation, while camphor inhibition destroys this process.

### 3.3. Camphor Effect on the Physiological State of Potato Tuber

Correlation analysis between the transcription and proteome of D vs. C group showed KEGG pathways of phenylpropanoid biosynthesis to be the most significant correlation, including eight up-regulated peroxidases exhibiting the same trends at the transcriptional level and in protein abundance. In potato, peroxidase/H_2_O_2_-mediated processes have been implicated in suberized cell wall polymerization [37]. Notably, two anionic peroxidases (PGSC0003DMT-400057521 and PGSC0003DMT400057522) showed increased transcriptional levels and protein abundance in the S vs. C groups and D vs. C groups. The results suggest that these two proteins were related to bud growth, which was not affect by camphor inhibition.

The EXORDIUM-LIKE1 protein was identified as a BR-up-regulated gene, and overexpression of this gene showed increased vegetative growth compared to wild-type plants in Arabidopsis, resembling the growth phenotype resulting from BR [38]. However, in the present study, this protein was increased at both the transcriptomics level and in proteomics abundance after camphor inhibition, with increased abundance in the D vs. S groups and up-regulated transcription levels in the S vs. C groups. Thus, we propose that this gene had different physiological functions in tuber, unlike in Arabidopsis. Thus, additional studies are needed to determine the function of this gene. Xyloglucan endotransglucosylase/hydrolase 1 and subtilisin-like protease SBT1.7 showed increased gene expression levels in the dormancy vs. camphor inhibition groups, while xyloglucan endotransglucosylase/hydrolase 1 showed the same expression trend in the D vs. S groups, and subtilisin-like protease SBT1.7 only showed increased protein abundance in the D vs. S groups. These two proteins have been implicated in cell wall synthesis in tomato and Arabidopsis [35,39]. Moreover, the data in the present study showed that camphor had no effect on cell wall synthesis during dormancy, consistent with physiological data.

The phosphoinositide phospholipase C2-like protein showed decreased expression in the transcription level and protein abundance in the D vs. C groups, while Stplc2 was up-regulated after wilting by severe short-term drought compare to untreated plants [40]. The down-regulation of phosphoinositide phospholipase C2 perhaps contributed to the reduction in water loss after camphor inhibition. In the present study, the buds of tubers became black and died after camphor inhibition for 50 days, and a protein in our study related to programmed cell death in Arabidopsis [41], was concomitantly up-regulated and showed increasing protein abundance. Thus, this protein may have function in bud necrosis after camphor inhibition.

### 3.4. Analysis on Uncoupling of Transcript Level and Protein Abundances

In addition to the same variation of transcripts level and protein abundances, there were many genes of which transcripts and protein variation trends were inversely correlated in different combinations. Previous research have also demonstrated that the degree of correlation between the transcriptome and the proteome is generally low (27–40%) [42]. Glutathione S-transferase plays an important role in plant defence against reactive oxygen damage and improving the repair ability of cell membrane [43]. The increase expression of glutathione S-transferase gene was a normal response to camphor inhibition stress in S vs. C, while the protein abundance was decreased. We speculated that the cell had been hurt and this affected protein synthesis and then exacerbated cell membrane damage, which was consistent with the damage of bud epidermis observed in Figure 1A. Aquaporin PIP2 was involved in the adjustment of plant water balance in response to changing environmental conditions, aquaporin PIP2;7 in *Arabidopsis* was found to be regulated at transcriptional and post-translational level under salt stress [44]. This gene remained unchanged at the transcriptional level, but showed decreased protein abundance in the S vs. C and the D vs. C groups. Hence, this protein was possibly degraded after camphor inhibition to limit water loss, and therefore, the tubers retained more moisture compared to the sprouting tuber (Figure 1C). Heat shock protein HSP20s assisted protein folding and maintained protein function stability under abiotic stress, participating in folding, transport and assembly of nascent polypeptide [45]. During tuber sprouting, increasing transcript and decreasing protein abundance of HSP20s may be caused by low translation efficiency or protein degradation.

Hu et al. also revealed poor correlations between mRNA and protein ratios in comparative proteomic and transcriptomic profiling of developing fibres between an elite cultivar and a wild variety [46]. In this experiment, aspartic proteinase nepenthesin (in S vs. C) and translocator protein (in D vs. C) were down-regulated at the transcriptional level while protein abundance increased. Aspartic proteinase nepenthesin genes were widely expressed in leaves, stems, seeds of *Arabidopsis*, suggesting multiple functions of the corresponding proteases, such as programmed cell death, disease and stress resistance, and leaf senescence [47]. AtTSPO (Tryptophan-rich sensory protein, a translocator protein) was induced by abiotic stresses and plant stress hormone abscisic acid (ABA) [48]. High abundance of AtTSPO suppressed aquaporin function at the plasma membrane to keep water content [49]. A possible explanation for uncoupling of transcript and protein abundances was that transcription level change was fast, but protein translation and modification lingered relatively, so after the changes of transcription level tended to be stable, the differences in protein abundance appeared [50]. Furthermore, at present, due to limitations of experimental techniques, DEGs and DAPs had only a small amount of overlap.

In summary, transcription and proteomics analysis of camphor regulating potato to sprouting revealed that camphor inhibited hormone synthesis and signal transduction, changing the relative gene expression levels and protein abundance, particularly of GA, BR and ETH (Ethylene), and promoted programmed cell death similarly to in pathogen defence. These processes lead to bud necrosis and prolong the storage date (Figure 9). It is vital to determine the flexible inhibitory action of camphor in potato tuber sprouting and reveal a mechanism model network relating to morphology, physiology, transcriptome, and proteomics. The study provides a novel insight into the development of new sprout inhibitors from aromatic plants. Additional studies using transgenic technology are needed to identify important gene functions in potato dormancy and sprouting in the future.

## 4. Materials and Methods

### 4.1. Plant Material Treatment and Physiological Index Analysis

Short-term dormancy potato cv. “Favorita” pre-basic seed tubers were obtained after harvest from the Potato Research and Development Centre at the College of Agronomy, Sichuan Agricultural University.

The tubers underwent wound healing for 2 weeks at room temperature in the dark after harvest, and subsequently, tubers weighing approximately 7–10 g were selected for study. Minitubers (1.6 kg) were placed in boxes (8 L) and maintained in the dark at 23 ± 2 °С. Camphor was placed on gauze and applied to the tubers in the form of vapour. Gauze containing camphor was positioned at the bottom of the boxes to avoid direct physical contact with the tubers. The content of camphor (≥98%, Sigma Aldrich Inc., Milwaukee, CA, USA) was 0.2 g/L. A control experiment was conducted under the same conditions. After each treatment, sprout length and weight loss were measured every 15 d from the beginning of the experiment. Tubers with at least one sprout longer than 2 mm were considered to sprouting. Tuber slides of the bud eye regions were examined at 4–8 × magnification using a stereomicroscope (OLYMPUS SZ51) with a camera. After storage for 110 d, the treated tubers were moved to ventilation conditions to observe sprouting recovery and growth. To analyse the concentrations of sucrose and fructose, tuber slices were maintained for 20 min at 105 °С, dried to a constant weight at 70 °С, and ground through a 150-μm aperture sieve to obtain a powder. The bud eye regions were obtained using a plastic pipe (5 mm diameter and 10 mm length) and subsequently used to analyse polyphenol oxidase (PPO) activity. The sucrose and fructose contents and PPO activity were measured as previously described [51,52].

### 4.2. Library Preparation and Sequencing

**Figure 10:**
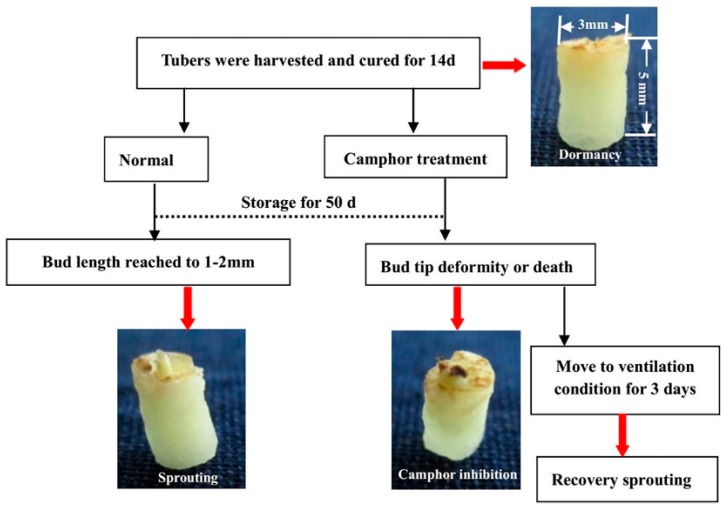
Treatment schematics for the experimental samples are listed. Red arrows represent the four types of samples.

The dormancy, sprouting, camphor inhibition and recovery sprouting samples were collected by obtaining bud eye regions using a plastic pipe (5 mm diameter and 10 mm length) and subsequently frozen in liquid nitrogen, followed by storage at −80 °С. The treatment process for the four samples is shown (Figure 10). RNA was isolated using TRIzol (Invitrogen, Carlsbad, CA, USA). Total mRNA was enriched using oligo (dT) magnetic beads. First-strand cDNA (Complementary DNA) was synthesized using random hexamer primers, and double-stranded DNA was purified using the QiaQuick PCR extraction kit (QIAGEN, Duesseldorf, Germany). The required fragments were purified using agarose gel electrophoresis and enriched via PCR. The completed library products were subjected to sequencing analysis using the Illumina HiSeqTM 2000 (Illumina, San Diego, CA, USA). The library construction and sequencing was performed at Beijing Genomics Institute (BGI), China.

### 4.3. Identification of Different Expression Genes and Bioinformatics Analysis

The clean tags were mapped to the potato reference genome and genes available at http://potato.plantbiology.msu.edu/pgsc_download.shtml [53] using the alignment software SOAPaligner/soap2(BGI). Only tags with perfect or two mismatches were annotated based on the reference genes. The expression level of each gene was estimated based on the frequency of tags and normalized to reads per Kb per million reads (RPKM) [54]. The tag frequency in each differentially expressed gene (DEG) library was statistically analysed. The threshold of the p-value in multiple tests was determined using the false discovery rate (FDR). A combination of FDR < 0.001 and the absolute value of log2 Ratio ≥ 1 was used as the threshold to judge the significance of gene expression differences. Gene ontology (GO) was used to assay the functional categories of the differentially expressed genes. Kyoto encyclopaedia of genes and genomes (KEGG) pathway enrichment was performed to identify significantly enriched metabolic pathways.

### 4.4. Quantitative RT-PCR

RNA was converted to cDNA using a RevertAid First-Strand cDNA Synthesis Kit(Invitrogen, Carlsbad, CA, USA). qRT-PCR was conducted in a 25 µL total volume containing 10.5 µL of RNase-Free ddH_2_O, 12.5 µL of 2 × SGExcel FastSYBR Mixture, 0.5 µL of Forward Primer, 0.5 µL of Reverse Primer, and 1.0 µL of cDNA. The thermal cycling conditions were 95 °С for 20 s, followed by 40 cycles at 95 °С for 3 s and 58–60 °С for 30 s. Melting curve analysis was conducted at 95 °С for 10 s, 65 °С for 5 s, and 95 °С for 5 s. The PCR reactions were separated using a Bio-Rad MiniOption System(Bio-Rad Laboratories, Hercules, CA, USA) and analysed using Bio-Rad CFX Connect(Bio-Rad Laboratories) and Excel software to determine the 2^−ΔΔCt^ values [55]. Three biological replicates and three technical replicates were analysed for each sample. The primers and fluorescent dyes (SGExcel FastSYBR Mixture) were purchased from Sangon Biotech (Shanghai, China). Elongation factor 1 alpha-like (EF1αL) was selected as a reference. The primer sequences used in qRT-PCR are shown (Table S9).

### 4.5. Protein Extraction, Digestion, and iTRAQ Labelling

Crude proteins were extracted from the four potato tuber samples, and the protein concentrations were measured using a Bio-Rad Protein Assay Kit. The quality of the proteins was assessed using sodium dodecyl sulphate polyacrylamide gel electrophoresis. Approximately 100 µg of protein for each sample was digested with trypsin (Promega, Madison, WI, USA) at an enzyme-to-substrate ratio of 1:50 for 12 h at 37 °С. After trypsin digestion, peptides were dried via vacuum centrifugation and reconstituted in 0.5 M of TEAB (Triethyl ammonium bicarbonate). The iTRAQ labelling procedure was performed according to the manufacturer’s instructions for 8-plex iTRAQ (Applied Biosystems, Foster City, CA, USA). In each group, the peptides were labelled with different iTRAQ tags after digestion. The mixture of iTRAQ-labelled samples was pooled, dried via vacuum centrifugation (Speed-Vac, Savant), and fractionated using strong cationic exchange (SCX) chromatography (Phenomenex, Guangzhou, China).

### 4.6. Liquid Chromatography-Mass Spectrometry and Database Search

The samples were fractionated using high pH reverse-phase HPLC with an Agilent 300 Extend C18 column (particles 5 μm, ID 4.6 mm, length 250 mm). Subsequently, the peptides were combined into 18 fractions and dried via vacuum centrifugation. The dried peptides were dissolved in 0.1% FA (formic acid) and directly loaded onto a reversed-phase pre-column (Acclaim PepMap 100, Thermo Fisher Scientific, Massachusetts, CA, USA). Peptide separation was performed using a reversed-phase analytical column (Acclaim PepMap RSLC, Thermo Scientific). The resulting peptides were analysed using a Q ExactiveTM plus Hybrid Quadrupole-Orbitrap Mass Spectrometer (Thermo Fisher Scientific). The resulting MS/MS data were processed using the Mascot search engine V.2.3.0 (Matrix Science Inc., Boston, CA, USA). The STRING database version 10.0 was used to analyse the protein–protein interaction networks.

### 4.7. Statistical Analysis

All data were subjected to unpaired Student’s t test at levels of *p* ≤ 0.01 and *p* ≤ 0.05. Data are shown as the means ± SE (*n* = 3), and *n* represents the biological replicates. Excel 2010 (Microsoft Corporation, Redmond, WA, USA) and SPSS14.0 software (IBM, New York, NY, USA) were used for data statistics.

## Figures and Tables

**Table 1:** Yield comparison per pot of recovery sprout tubers.

Seed Tuber	Number of Seeding	Number of Tubers	Tuber Weight (g)
Normal	1.33 ± 0.49 B	2.40 ± 0.83 B	24.16 ± 5.43 a
Recovery sprouting	2.86 ± 1.61 A	4.14 ± 1.75 A	19.38 ± 6.11 b

Data in the table show the means ± SD of 15 pots. Values followed by different capital or lowercase letters in the vertical line represent significant differences at 0.01 or 0.05 probability levels between normal condition and recovery sprouting.

**Table 2:** Five typical expression profiles of DEGs in four samples. D: dormancy; S: sprouting: C: camphor inhibition; R: recovery sprouting.

	Gene ID	D	S	C	R	Functional Annotation
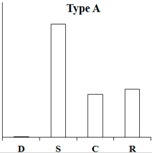	N16546	0.01	50.35	19.09	21.41	GDSL(Gly-Asp-Ser-Leu)-like lipase/acylhydrolase family protein
	N55828	0.01	23.67	12.36	18.00	E3 ubiquitin-protein ligase RMA1H1
	N88323	0.01	15.47	13.51	18.48	Aspartic proteinase nepenthesin-1
	N30676	11.32	1242.54	500.20	886.62	2-oxoglutarate-dependent dioxygenase
	N05343	0.98	251.85	155.24	183.27	Pectinesterase
	N30650	1.42	209.91	103.84	159.95	β-solanine/β-chaconine rhamnosyltransferase
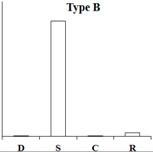	N76367	0.12	34.15	0.01	1.09	Proline-rich protein 1
	N83077	0.24	20.90	1.10	13.96	Gip1
	N24372	0.01	5.78	0.01	3.23	Hypothetical gene of unknown function
	N23187	0.01	19.75	1.42	16.42	Xyloglucan-specific endoglucanase inhibitor 4
	N30708	0.01	24.63	2.95	6.81	GATA-type transcription factor 4-like or AG-motif binding protein-3
	N81314	3.23	40.78	6.27	9.18	Endo-1,4-beta-glucanase
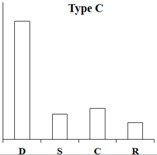	N37745	4.71	0.01	0.01	0.30	Conserved gene of unknown function
	N53578	4.23	0.58	0.59	1.87	Conserved gene of unknown function
	N41670	4.69	0.69	1.13	1.20	Serine/threonine-protein kinase SAPK1
	N69883	43.11	9.27	11.40	6.10	Protein phosphatase 2c
	N07511	35.47	12.23	8.31	6.28	Dehydration-responsive element binding protein
	N04046	2353.85	939.44	881.62	1155.99	Snakin-2
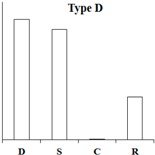	N24747	5.25	4.81	0.01	1.86	Hypothetical gene of unknown function
	N05722	5.88	6.55	0.96	2.49	Hypothetical gene of unknown function
	N35281	5.21	4.76	0.65	2.00	UDP(Uridine diphosphate)-sugar:glycosyltransferase
	N76038	16.88	12.91	2.36	8.70	Hypothetical gene of unknown function
	N40696	18.21	12.56	3.12	5.88	Leucine-rich repeat resistance protein
	N47882	14.98	10.02	2.70	3.06	Hypothetical gene of unknown function
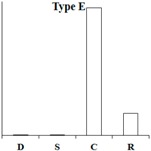	N47338	0.01	0.01	23.80	4.11	Wound-induced protein
	N52057	0.01	0.15	9.11	0.44	WRKY DNA binding protein
	N26225	0.38	0.87	26.82	10.63	Conserved gene of unknown function
	N07870	0.90	3.96	40.06	6.74	Osmotin
	N47343	5.63	9.02	89.09	26.79	Wound-induced protein
	N03936	7.76	43.00	420.05	279.97	Pathogenesis-related protein STH-2 or TSI-1 protein

N = PGSC0003DMT4000.

## Supplementary Materials

Supplementary materials can be found at www.mdpi.com/1422-0067/18/11/2280/s1.

## References

[1] Moll A. (1985). Effect of the physiological age of seed tubers on growth and yield of potato cultivars of different maturity classes. Potato Res..

[2] Hartmann A., Senning M., Hedden P., Sonnewald U., Sonnewald S. (2011). Reactivation of meristem activity and sprout growth in potato tubers require both cytokinin and gibberellin. Plant Physiol..

[3] Daniels-Lake B.J., Pruski K., Prange R.K. (2011). Using ethylene gas and chlorpropham potato sprout inhibitors together. Potato Res..

[4] Campbell M.A., Gleichsner A., Hilldorfer L., Horvath D., Suttle J. (2012). The sprout inhibitor 1,4-dimethylnaphthalene induces the expression of the cell cycle inhibitors KRP1 and KRP2 in potatoes. Funct. Integr. Genom..

[5] Weerd D.J.W., Thornton M.K., Shafii B. (2010). Sprout suppressing residue levels of 1,4-dimethylnaphthalene (1,4DMN) in potato cultivars. Am. J. Potato Res..

[6] Gómez-Castillo D., Cruz E., Iguaz A., Arroqui C., Vírseda P. (2013). Effects of essential oils on sprout suppression and quality of potato cultivars. Postharvest Biol. Technol..

[7] Hartmans K.J. (1995). The use of carvone in agriculture: Sprout suppression of potatoes and antifungal activity against potato tuber and other plant diseases. Ind. Crops Prod. Appl..

[8] Oosterhaven K., Poolman B., Smid E.J. (1995). S-carvone as a natural potato sprout inhibiting, fungistatic and bacteristatic compound. Potato Res..

[9] Bamnolker T.P., Dudai N., Fischer R., Belausov E., Zemach H., Shoseyov O. (2010). Mint essential oil can induce or inhibit potato sprouting by differential alteration of apical meristem. Planta.

[10] Zhang G., Guo G., Hu X., Zhang Y., Li Q., Li R. (2010). Deep RNA sequencing at single base-pair resolution reveals high complexity of the rice transcriptome. Genome Res..

[11] Cheng Y., Cao L., Wang S., Li Y., Shi X., Liu H., Li L., Zhang Z., Fowke L.C., Wang H. (2013). Down regulation of multiple CDK inhibitor ICK/KRP genes upregulates the E2F pathway and increases cell proliferation, and organ and seed sizes in Arabidopsis. Plant J..

[12] Liu B., Zhang N., Wen Y., Jin X., Yang J., Si H. (2015). Transcriptomic changes during tuber dormancy release process revealed by RNA sequencing in potato. J. Biotechnol..

[13] Wilm M. (2009). Quantitative proteomics in biological research. Proteomics.

[14] Yang Y., Qiang X., Owsiany K., Zhang S., Thannhauser T.W., Li L. (2011). Evaluation of different multidimensional LC-MS/MS pipelines for isobaric tags for relative and absolute quantitation (iTRAQ)-based proteomic analysis of potato tubers in response to cold storage. J. Proteome Res..

[15] Liu B., Zhang N., Zhao S., Chang J., Wang Z., Zhang G. (2015). Proteomic changes during tuber dormancy release process revealed by iTRAQ quantitative proteomics in potato. Plant Physiol. Biochem..

[16] Mani F., Bettaieb T., Doudech N., Hannachi C., Institut H.A., Chott-Mariem P.O.B. (2014). Physiological mechanisms for potato dormancy release and sprouting a review. Afr. Crop Sci. J..

[17] Sonnewald S., Sonnewald U. (2014). Regulation of potato tuber sprouting. Planta.

[18] Wilson N.S., Rueschhoff E.E., Bhatti H., Franks R.G. (2010). Synergistic disruptions in seuss cyp85A2 double mutants reveal a role for brassinolide synthesis during gynoecium and ovule development. BMC Plant Biol..

[19] Reinecke D.M., Wickramarathna A.D., Ozga J.A., Kurepin L.V., Jin A.L., Good A.G. (2013). Gibberellin 3-oxidase gene expression patterns influence gibberellin biosynthesis, growth, and development in pea. Plant Physiol..

[20] Quan J. (2013). The Role of Terpenoids in Plants and Its Application. Bot. Res..

[21] Yang X., Wang S.S., Wang M., Qiao Z., Bao C.C., Zhang W. (2014). Arabidopsis thaliana calmodulin-like protein CML24 regulates pollen tube growth by modulating the actin cytoskeleton and controlling the cytosolic Ca^2+^ concentration. Plant Mol. Biol..

[22] Wu X., Qiao Z., Liu H., Acharya B.R., Li C., Zhang W. (2017). CML20, an Arabidopsis Calmodulin-like Protein, Negatively Regulates Guard Cell ABA Signaling and Drought Stress Tolerance. Front. Plant Sci..

[23] Liu B., Zhang N., Wen Y., Si H., Wang D. (2012). Identification of differentially expressed genes in potato associated with tuber dormancy release. Mol. Biol. Rep..

[24] Petrovova M., Tkadlec J., Dvoracek L., Streitova E., Licha I. (2014). NAD(P)H-hydrate dehydratase- a metabolic repair enzyme and its role in Bacillus subtilis stress adaptation. PLoS ONE.

[25] Shi L., Olszewski N.E. (1998). Gibberellin and abscisic acid regulate GAST1 expression at the level of transcription. Plant Mol. Biol..

[26] Wang L., Wang Z., Xu Y., Joo S.H., Kim S.K., Xue Z. (2009). OsGSR1 is involved in crosstalk between gibberellins and brassinosteroids in rice. Plant J..

[27] Campbell M.A., Gleichsner A., Alsbury R., Horvath D., Suttle J. (2010). The sprout inhibitors chlorpropham and 1,4-dimethylnaphthalene elicit different transcriptional profiles and do not suppress growth through a prolongation of the dormant state. Plant Mol. Biol..

[28] Fan Z.J., Liu X.F., Liu F.L., Bao L.L., Zhang Y.G. (2005). Progress of researches on induced resistance of plant activator. Acta Phytophylacica Sin..

[29] Marineau C., Matton D.P., Brisson N. (1987). Differential accumulation of potato tuber mRNAs during the hypersensitive response induced by arachidonic acid elicitor. Plant Mol. Biol..

[30] Constabel C.P., Brisson N. (1992). The defense-related STH-2 gene product of potato shows race-specific accumulation after inoculation with low concentrations of Phytophthora infestans zoospores. Planta.

[31] Matton D.P., Prescott G., Bertrand C., Camirand A., Brisson N. (1993). Identification of cis-acting elements involved in the regulation of the pathogenesis-related gene STH-2 in potato. Plant Mol. Biol..

[32] Constabel C.P., Bertrand C., Brisson N. (1993). Transgenic potato plants overexpressing the pathogenesis-related STH-2 gene show unaltered susceptibility to Phytophthora infestans and potato virus X. Plant Mol. Biol..

[33] Rehman K.U.M., Sun L., Li C.X., Faheem M., Wang W., Tao J.M. (2017). Comparative RNA-seq based transcriptomic analysis of bud dormancy in grape. BMC Plant Biol..

[34] Ibañes M., Fabregas N., Chory J., Cano-Delgado A.I. (2009). Brassinosteroid signaling and auxin transport are required to establish the periodic pattern of Arabidopsis shoot vascular bundles. Proc. Natl. Acad. Sci. USA.

[35] Albert M., Werner M., Proksch P., Fry S.C., Kaldenhoff R. (2004). The cell wall-modifying xyloglucan endotransglycosylase/hydrolase LeXTH1 is expressed during the defence reaction of tomato against the plant parasite Cuscuta reflexa. Plant Biol..

[36] Singh R.K., Ali S.A., Nath P., Sane V.A. (2011). Activation of ethylene-responsive p-hydroxyphenylpyruvate dioxygenase leads to increased tocopherol levels during ripening in mango. J. Exp. Bot..

[37] Kolattukudy P.E. (1980). Biopolyester membranes of plants: Cutin and suberin. Science.

[38] Garcia CD., Mazuch J., Altmann T., Mussig C. (2004). EXORDIUM regulates brassinosteroid-responsive genes. FEBS Lett..

[39] Rautengarten C., Usadel B., Neumetzler L., Hartmann J., Bussis D., Altmann T. (2008). A subtilisin-like serine protease essential for mucilage release from Arabidopsis seed coats. Plant J..

[40] Kopka J., Pical C., Gray J.E., Muller-Rober B. (1998). Molecular and enzymatic characterization of three phosphoinositide-specific phospholipase C isoforms from potato. Plant Physiol..

[41] Swidzinski J.A., Leaver C.J., Sweetlove L.J. (2004). A proteomic analysis of plant programmed cell death. Phytochemistry.

[42] Muers M. (2011). Gene expression: Transcriptome to proteome and back to genome. Nat. Rev. Genet..

[43] Armstrong R.N. (1997). Structure, catalytic mechanism, and evolution of the glutathione transferases. Chem. Res. Toxicol..

[44] Pou A., Jeanguenin L., Milhiet T., Batoko H., Chaumont F., Hachez C. (2016). Salinity-mediated transcriptional and post-translational regulation of the Arabidopsis aquaporin PIP2;7. Plant Mol. Biol..

[45] Klein R.D., Chidawanyika T., Tims H.S., Meulia T., Bouchard R.A., Pett V.B. (2014). Chaperone function of two small heat shock proteins from maize. Plant Sci..

[46] Hu G.J., Koh J., Yoo M.J., Grupp K., Chen S., Wendel J.F. (2013). Proteomic profiling of developing cotton fibers from wild and domesticated Gossypium barbadense. New Phytol..

[47] Takahashi K., Niwa H., Yokota N., Kubota K., Inoue H. (2008). Widespread tissue expression of nepenthesis-like aspartic protease genes in Arabidopsis thaliana. Plant Physiol. Biol..

[48] Guillaumot D., Guillon S., Deplanque T., Vanhee C., Gumy C., Masquelier D., Morsomme P., Batoko H. (2009). The Arabidopsis TSPO-related protein is a stress and abscisic acid-regulated, endoplasmic reticulum-Golgi-localized membrane protein. Plant J..

[49] Cui N., Song Z., Yang B., Fan L.M. (2016). AtTSPO, a translocator protein, in stress responses in Arabidopsis. Environ. Exp. Bot..

[50] Chen J., Liu S.S., Kohler A., Yan B., Luo H.M., Chen X.M., Guo S.X. (2017). iTRAQ and RNA-Seq analyses provide new insights into regulation mechanism of symbiotic germination of *Dendrobium officinale* seeds (Orchidaceae). J. Proteome Res,.

[51] Zhang K., Wu Z., Tang D., Luo K., Lu H., Liu Y. (2017). Comparative transcriptome analysis reveals critical function of sucrose metabolism related-enzymes in starch accumulation in the storage root of sweet potato. Front. Plant Sci..

[52] Sun J., You X., Li L., Peng H., Su W., Li C. (2011). Effects of a phospholipase D inhibitor on postharvest enzymatic browning and oxidative stress of litchi fruit. Postharvest Biol. Technol..

[53] The Potato Genome Sequencing Consortium (2011). Genome sequence and analysis of the tuber crop potato. Nature.

[54] Mortazavi A., Williams B.A., McCue K., Schaeffer L., Wold B. (2008). Mapping and quantifying mammalian transcriptomes by RNA-Seq. Nat. Methods.

[55] Livak K.J., Schmittgen T.D. (2001). Analysis of relative gene expression data using real-time quantitative PCR and the 2(-Delta Delta C(T)) Method. Methods.

