# Supplementary material for: Comparative Morphology, Transcription, and Proteomics Study Revealing the Key Molecular Mechanism of Camphor on the Potato Tuber Sprouting Effect
Source: Int J Mol Sci. 2017 Oct 30;18(11):2280. doi: 10.3390/ijms18112280 (PMC5713250; doi:10.3390/ijms18112280)
Supplement: Supplementary file 1 [file ijms-18-02280-s001.zip › Table S6.pdf]

Table S6. Primer sequences used in Quantitative RT-PCR for iTRAQ validation.

ID	Gene	Primer
PGSC0003DMT400006894	Uncharacterized protein	TGGTGGACAGAGTGAAGAA
		CGAGTGTAGCATCGGAGTT
PGSC0003DMT400003773	Pathogenesis-related protein STH-2-like	CAACGACCACAATTTCCC
		TCCAACACCACCATCTCC
PGSC0003DMT400039734	Protochlorophyllide reductase-like	GGGATGCCTAAGGAGAAT
		CGAACCGACAATGATGAG
PGSC0003DMT400042741	4-alpha-glucanotransferase	TTCTTCTGTAGCCCGTAT
		AACTAGGTATCCTCCAACTC
PGSC0003DMT400015734	Heat shock protein 90-6	CAGTTTGTTTCATTCCCTAT
		ATCTTGCCCTTCCTTATT
PGSC0003DMT400037918	Glutathione s-transferase	GCCCATTTAGTCACAGAG
		CAAGGAACTTAGCCCAGA
PGSC0003DMT400005104	Glutathione s-transferase	ATGGAAGAATGGACCTCA
		CCAAATGCTACGACGATC
PGSC0003DMT400042214	Ubiquitin-conjugating enzyme E2	GTTCAGGAGGATCTAGTGTT
		CAGAATTGTGAGGACCAA
PGSC0003DMT400090964	Protein transport protein Sec61	GTGGTAACGCTGTAGTAGGC
		GTAGTAACGAATCCGATGAAG
PGSC0003DMT400033585	14-3-3 protein	TTGAACAGAAGGAGGAAT
		GGTAGTAGCAGACGGAAC
PGSC0003DMT400018083	dnaJ protein homolog	TCAATACAAGGCGATAAATG
		GCTGCTCCTCGTGTCTCA
PGSC0003DMT400042739	Glycoside hydrolase	TTCTTCTGTAGCCCGTAT
		AACTAGGTATCCTCCAACTC
