# Supplementary material for: Comparative Morphology, Transcription, and Proteomics Study Revealing the Key Molecular Mechanism of Camphor on the Potato Tuber Sprouting Effect
Source: Int J Mol Sci. 2017 Oct 30;18(11):2280. doi: 10.3390/ijms18112280 (PMC5713250; doi:10.3390/ijms18112280)
Supplement: Supplementary file 1 [file ijms-18-02280-s001.zip › Table S9.pdf]

Table S9. Quantitative RT-PCR for RNA sequencing validation.

ID	Gene	Primer
PGSC0003DMT400014674	Elongation factor 1 alpha-like protein ***EF1αL***	CTTGTACACCACGCTAAGGAG
		GTCAATGCAAACCATTCCTTG
PGSC0003DMT400033236	4,4-dimethyl-9beta,19-cyclopropylsterol-4alpha-methyl oxidase ***SMO*1**	GAGAGAAACTGAGAATGGAGG
		GTGCTTGATAGAGAAACACAAC
PGSC0003DMT400020648	Cyclin D3 ***CYCD*3**	TGATAGGCTTAAACCTTGC
		TAACAGATGTAGCCACTGAC
PGSC0003DMT400064270	Peroxidase ***POD***	CACTGTCCCTGCTGTTC
		TCCTGCCAATGATAGATTA
PGSC0003DMT400023554	Wax-ester synthase / diacylglycerol O-acyltransferase ***WES***	GTGTTGACCCGATTACCCA
		CTTGTTTAGTGACAGCCTCTT
PGSC0003DMT400028227	Myb proto-oncogene transcription factor MYB48-like ***MYB*48**	GACGACACTTTGCACCAAC
		TCCAAGACAAGCCTACCTAAT
PGSC0003DMT400014568	General transcription factor IIE subunit 1-like ***TFIIE***	AGGAAGGAGGGAGCAAA
		AGGCAGCAATAAGAATGAG
PGSC0003DMT400004046	Snakin2 ***SN*2**	CTCCTTGCTCCTTCTCG
		TAGCAAGGGCAAGTCTCAG
PGSC0003DMT400019919	Kip-related protein 4 ***KRP*4**	AAAAGACAGAATCGGAGTT
		GTCCTCTCTCTACCATCAAT
PGSC0003DMT400056352	WRKY transcription factor 75 ***WRKY* 75**	CAGCATCATCGTCGTCAT
		CATTAGTCCCAAGAACCC
PGSC0003DMT400015017	Transcription factor ATR2-like ***ATR*2**	CTCATAGTGCGTCCTCCA
		AATCACACCTTCAGATCCAAT
PGSC0003DMG400001550	Pathogenesis-related protein STH-2 ***STH*-2** or TSl-1 protein	AAAACCAGGCATGGAACT
		ACGTGTAGACCTGATTCTTT
PGSC0003DMT400020263	Serine-rich protein ***SRP***	CCAAACACGGCACAAAGA
		GGGACAGTGAACGGAGAA
