# Supplementary figures and images for: Comparative Morphology, Transcription, and Proteomics Study Revealing the Key Molecular Mechanism of Camphor on the Potato Tuber Sprouting Effect
Source: Int J Mol Sci. 2017 Oct 30;18(11):2280. doi: 10.3390/ijms18112280 (PMC5713250; doi:10.3390/ijms18112280)

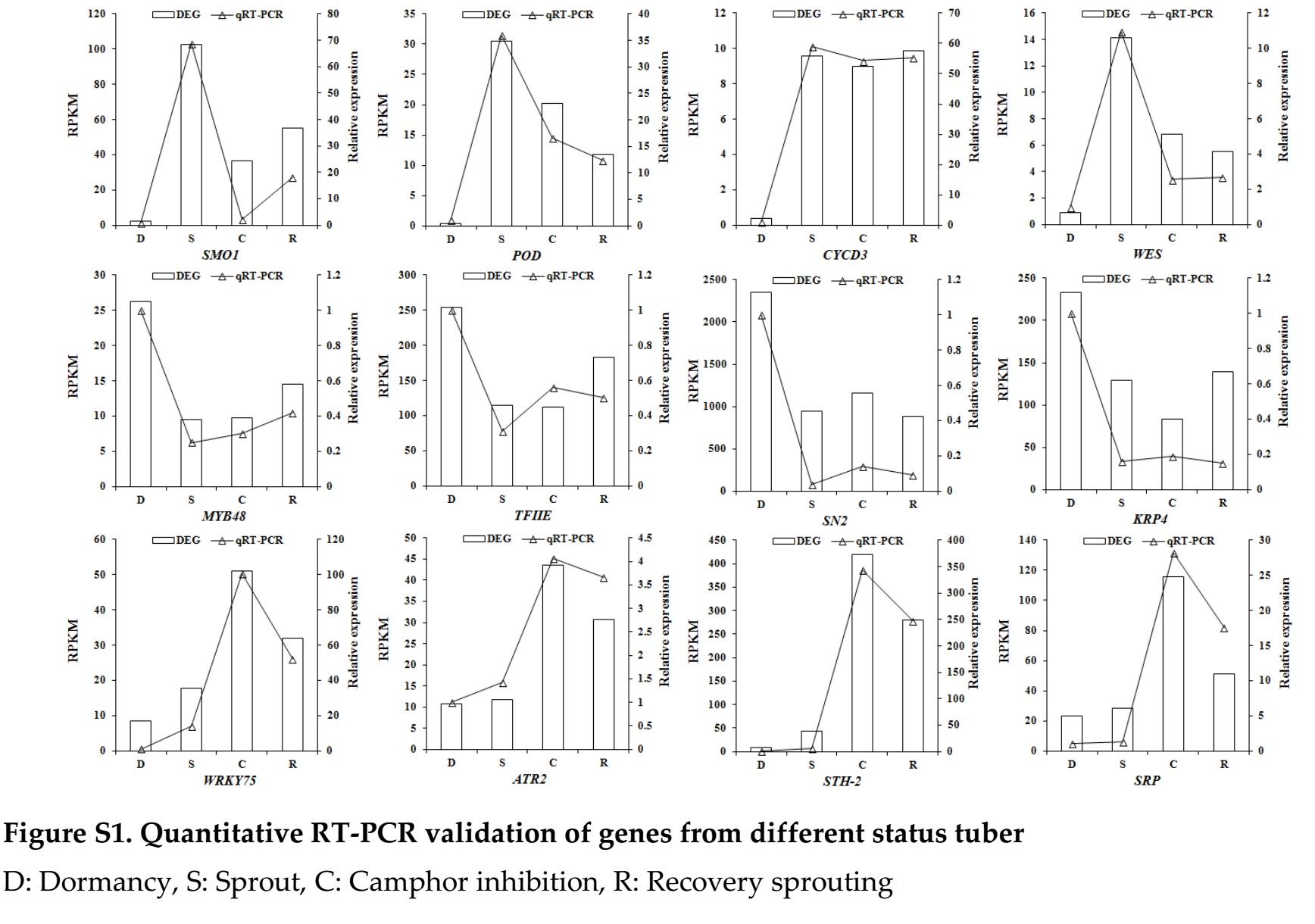

Supplement: Supplementary file 1 [file ijms-18-02280-s001.zip › Figure S1.jpg]

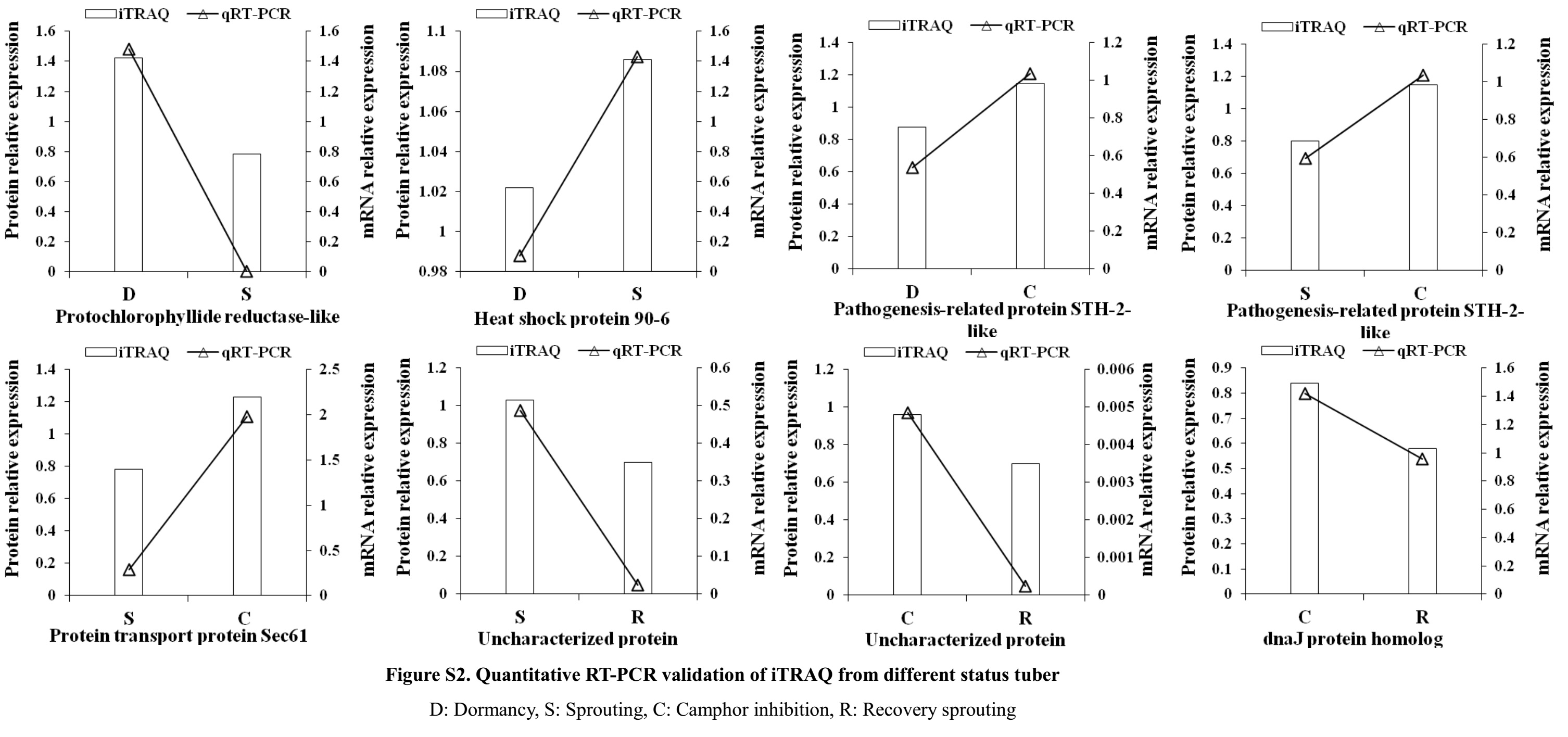

Supplement: Supplementary file 1 [file ijms-18-02280-s001.zip › Figure S2.jpg]

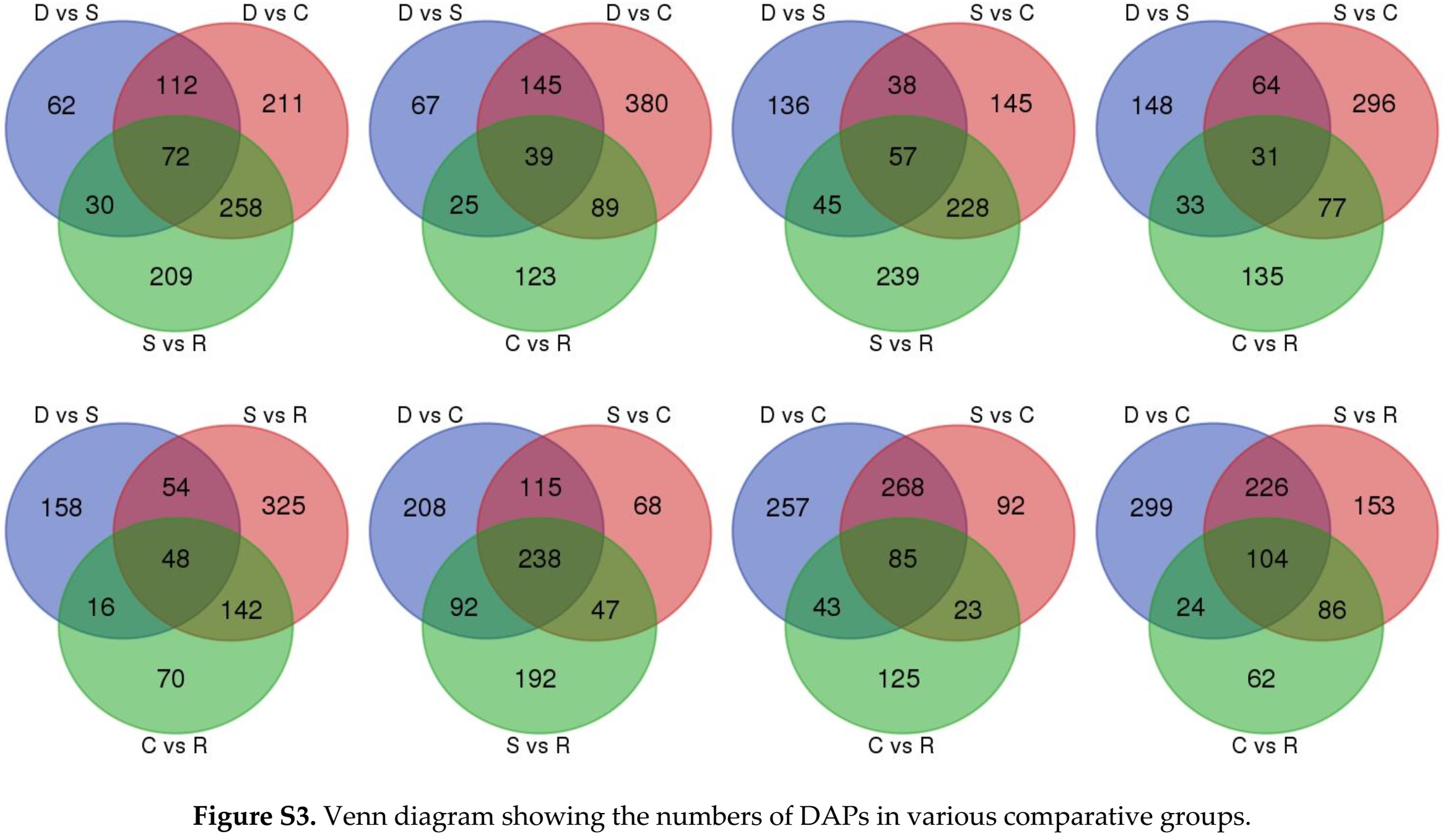

Supplement: Supplementary file 1 [file ijms-18-02280-s001.zip › Figure S3.jpg]

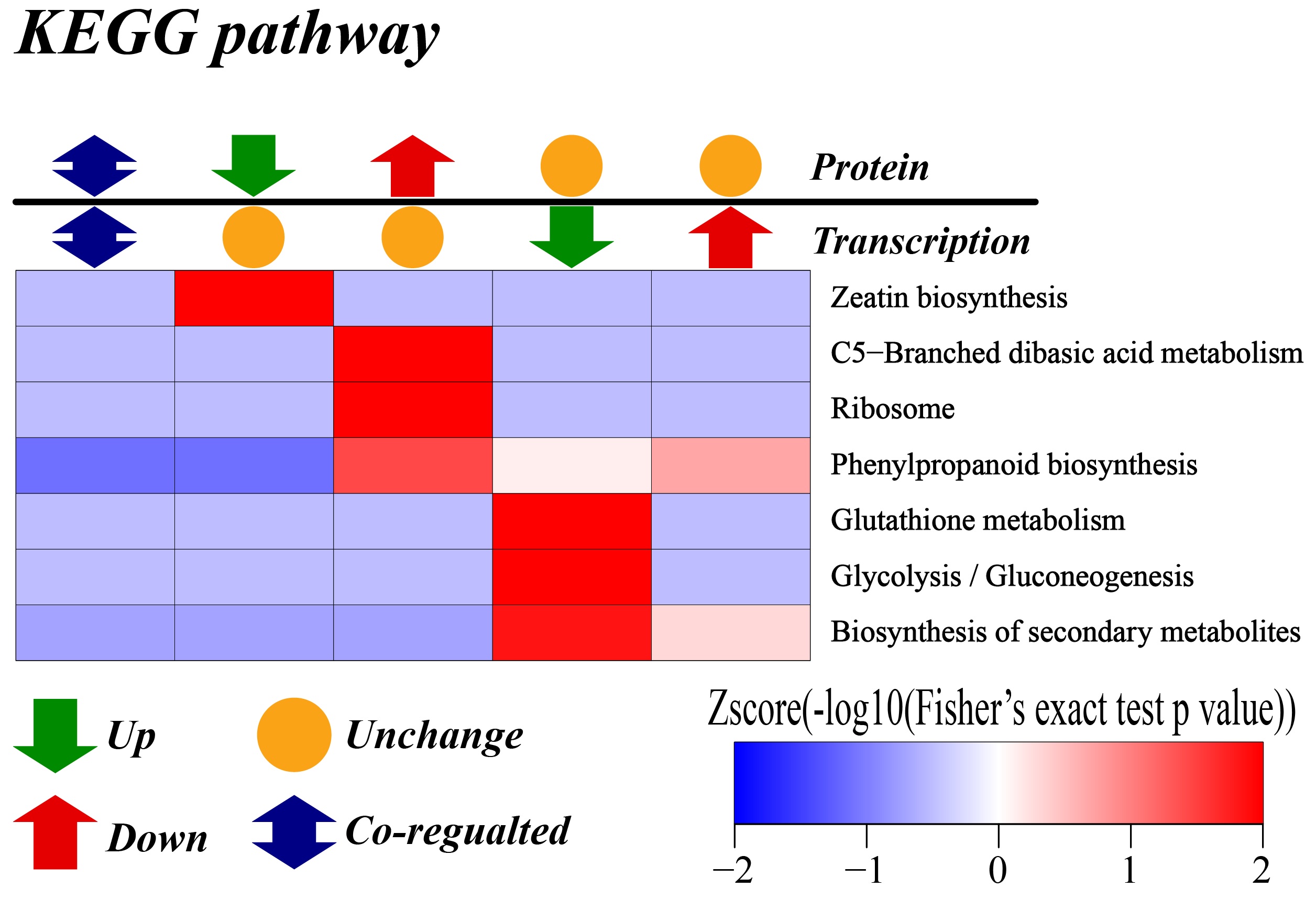

Supplement: Supplementary file 1 [file ijms-18-02280-s001.zip › Figure S4.jpg]

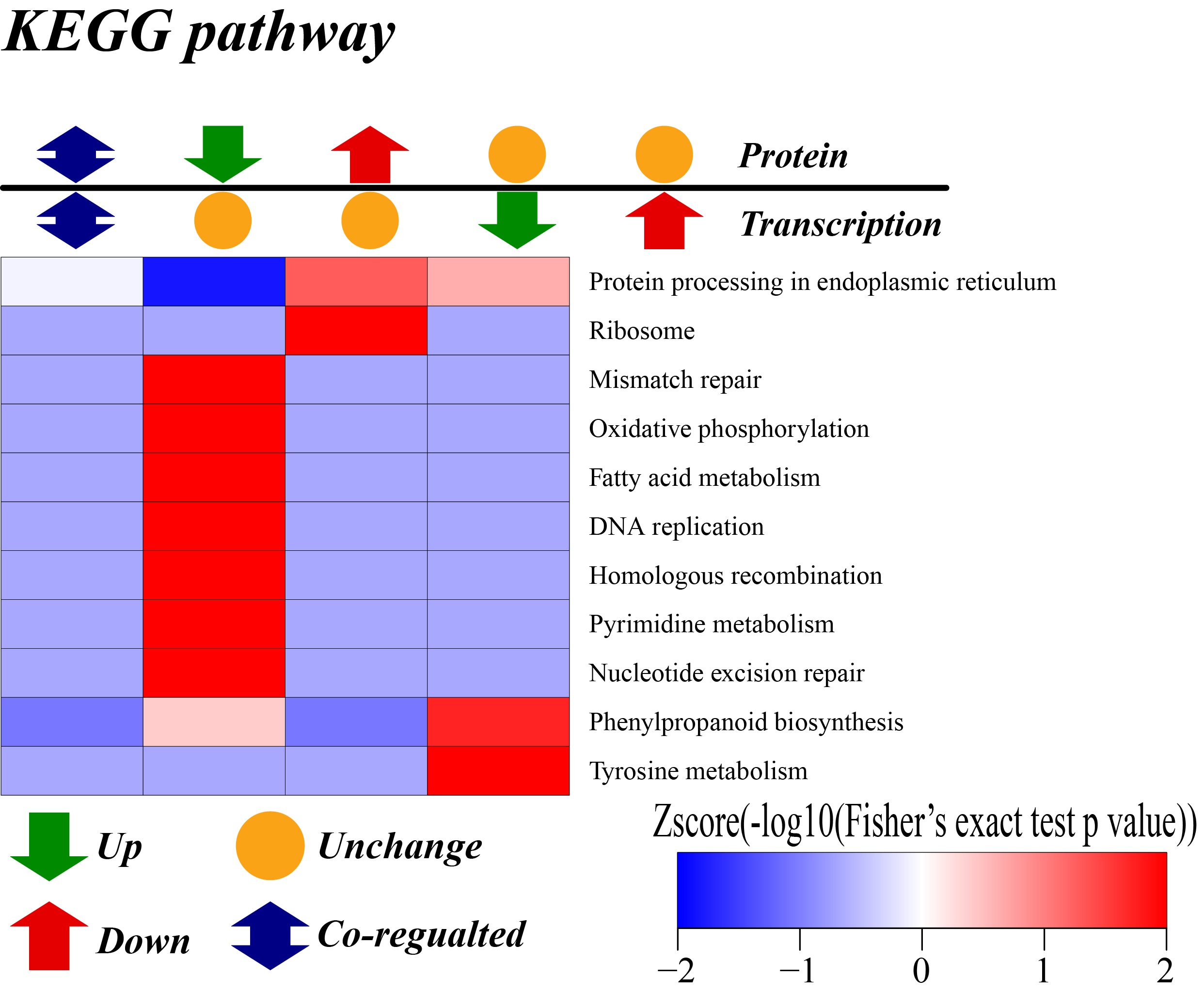

Supplement: Supplementary file 1 [file ijms-18-02280-s001.zip › Figure S5.jpg]
